# Pain in non-communicative older adults beyond dementia: a narrative review

**DOI:** 10.3389/fmed.2024.1393367

**Published:** 2024-08-15

**Authors:** Luca Tagliafico, Giada Maizza, Silvia Ottaviani, Mariya Muzyka, Federica Della Rovere, Alessio Nencioni, Fiammetta Monacelli

**Affiliations:** ^1^Geriatrics Clinic, Department of Internal Medicine and Medical Specialties (DIMI), University of Genoa, Genoa, Italy; ^2^IRCCS Ospedale Policlinico San Martino, Genoa, Italy

**Keywords:** pain, older adults, non-verbal, non-communicative, dementia and neurodegenerative disorders

## Abstract

Aging is associated with an increased risk of developing pain, especially in the presence of concurrent chronic clinical conditions. Similarly, multimorbidity can affect the perception and ability of older adults to appropriately respond to and communicate pain, and there is a clinical heterogeneity in the processing of painful sensations in different neurological conditions. The present narrative review is aimed at assessing the prevalent diseases associated with poor communication and pain in older adults, together with the available diagnostic instruments for the clinical assessment of pain in such a vulnerable population. Dementia was the most described pathology identified in the current literature associated with poor communication in older adults affected by pain, along with Parkinson’s disease and stroke. Notably, a common pattern of pain behaviors in these neurological disorders also emerged, indicating potential similarities in the clinical presentation and appropriate diagnostic workout. At the same time, there are many differences in the way patients express their pain according to their main neurological pathology. In addition to this, although a plethora of observation-based tools for pain in patients with dementia have been developed, there is no gold standard, and the clinical utility of such measurements is still largely unaddressed. Meanwhile, there is substantially no standardized observation-based tool for pain in non-communicative patients with Parkinson’s disease, and only a few for stroke. Overall, the present narrative review provides an update on the prevalent diseases beyond dementia associated with a communicative disability and a painful condition in older adults.

## Introduction

Aging is associated with an increased risk of developing pain, especially in the presence of multimorbidity and frailty (1). The high prevalence of chronic pain in both community-dwelling older persons and nursing home residents (2–4) is associated with unfavorable clinical outcomes, including poorer cognitive performance, reduced quality of life, depression, functional decline, disability, and social vulnerability (5, 6).

Similarly, with aging, there is a net increase in neurological conditions, especially in frail patients, that may impact communicative ability, with a higher likelihood of failure in the identification and appropriate management of pain in such vulnerable individuals (7, 8). Additionally, aging could bring changes in pain processing and communication, which can render pain assessment tools typically used for younger individuals unreliable (9). Finally, changes in pharmacokinetics and pharmacodynamics, coupled with the polypharmacotherapy often seen in older adult patients, increase the risk of adverse events from pain medications. This adds to the risk of underdiagnosis of pain, making its management even more complex (10).

To date, dementia is the key relevant clinical condition associated with communicative disabilities (11–15). Pain expressions in patients with dementia often take less obvious forms, such as confusion or social withdrawal, that are behavioral equivalents of pain in non-communicative patients. To overcome the limited diagnostic accuracy of pain self-report tools in non-verbal communicative persons, whose ability to respond to direct pain questioning is impacted, the American Geriatrics Society and the American Society for Pain Management Nursing (16–18) selected a list of behavioral pain indicators to develop reliable observational-based pain assessment tools. Namely, facial expressions, vocalizations, body movements, changes in interpersonal interactions, basal activities of daily living, and mental status have been reported to be the most sensitive indicators of pain in non-communicative older adults with dementia (19).

These effective measures have been implemented to recognize and treat pain in a timely manner for such patients. However, in an effort to build and refine clinical recommendations after two decades, no specific tool is considered the gold standard, and the assessment of pain or discomfort in non-communicative patients remains a major challenge. Pain communication in older adult patients can be complex owing to several factors, including cultural variables, apart from specific clinical morbid conditions (7).

Starting from this background, this narrative review is aimed at assessing the prevalent diseases associated with pain in non-communicative older adults, along with a brief overview of the available diagnostic instruments for the clinical assessment of pain in such a vulnerable population. Furthermore, by virtue of what was mentioned earlier, our review also aims, as an overall goal, to provide insights for further aspects to be researched or implemented from the current literature on the subject.

## Materials and methods

This review was based on a search in the MEDLINE, Scopus, and PEDro databases for articles in English published from January 1, 1990, to June 22, 2024, regarding the presence of pain in non-communicative older adults.

The Scale for the Assessment of Narrative Review Articles (SANRA) was used as a methodological guideline in conducting the narrative review (20). Briefly, the six items that form the scale are rated from 0 to 2, with 1 as an intermediate score. The maximal sum score is 12. The sum score of the scale is intended to measure the quality of a narrative review and covers the following topics: justification of the article’s importance for the readership (item 1), statement of the aims or formulation of questions (item 2), description of the literature search (item 3), referencing (item 4), scientific reasoning (item 5), and appropriate presentation of data (item 6). It represents a scale developed especially for the evaluation of narrative reviews by editors and peer reviewers. It is also used, as in our case, in the drafting phase of the article in order to make it as organic and rigorous as possible.

### Search terms

- Pain was defined as ‘an unpleasant sensory and emotional experience associated with actual or potential tissue damage’ or described in terms of such damage, according to the International Association of Pain (21). Based on the standard definition, ‘chronic pain is one such common ailment reported in older adults that also poses a significant economic burden on health care.’ Chronic pain is defined as pain that lasts for 3 to 6 months or more than expected (22). Chronic, acute, and/or breakthrough, musculoskeletal, neuropathic, ischaemic, mixed, and cancer pains were included in the search.
- Non-communicative or non-verbal persons were referred to as older adults with impaired ability to perceive, express, or verbally communicate pain or discomfort.
- The older adult term used in our literature search referred to all studies that included patients aged 65 years or older. In geriatrics, the categorization of aging is based on the following stratifications: young old (age: 65–74 years), old (75–84 years), and oldest old (≥85 years).
- The settings of care were community dwellings, nursing homes, hospitals, and transitional care units.

### Inclusion criteria

The inclusion criteria were all the above-mentioned keywords in all possible combinations. Retrospective, prospective cohort, observational, and interventional studies that evaluated at least 50 patients were included.

### Exclusion criteria

Abstracts, editorials, case studies, score creation studies, pilot studies, studies with fewer than 50 patients, and studies without a specific focus on older adults (i.e., adults aged <65 years or no data including old age participants); older adults with pre-existing intellectual disability; patients admitted to intensive care units; and older adults with disorders of consciousness were excluded.

The initial phase of article selection was conducted by the two co-first authors, who reached a common agreement on the chosen articles. In the subsequent phase, rather than extraction, the two aforementioned authors independently undertook separate tasks: one focused on pathologies associated with communication issues, while the other concentrated on methods for pain assessment within this context. A comprehensive evaluation of the findings was then conducted, drawing from the articles selected by other contributors to the review.

**Figure 1:**
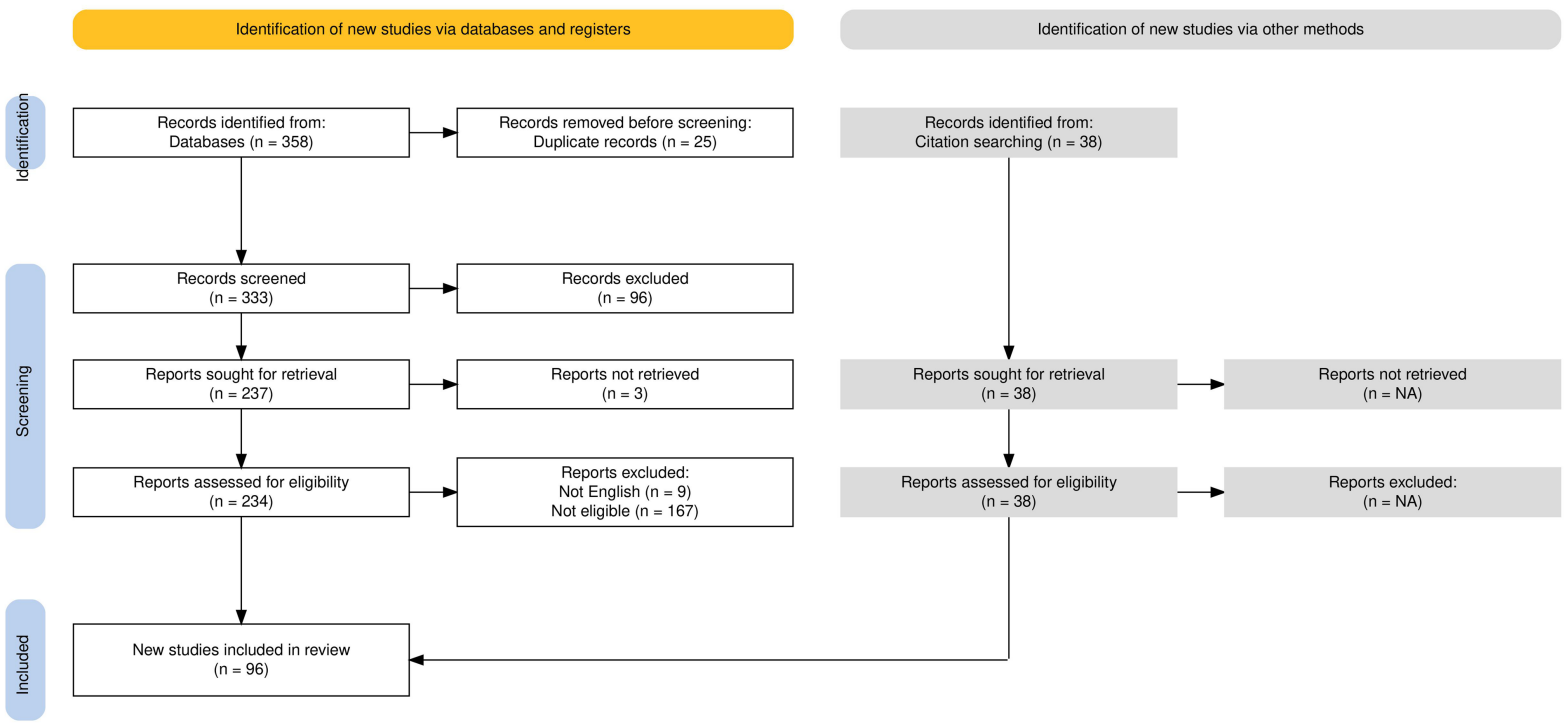
PRISMA flowchart for article selection.

Figure 1 illustrates the selection process through a PRISMA flowchart (23). We included 58 suitable studies from 358 articles initially identified in the selected databases, as well as 38 articles not present in the chosen databases but included in the references of the identified articles. In particular, of the 358 initially identified articles, 25 were removed because they were duplicates; 96 were excluded according to the previously explained criteria based on the title and the abstract; and 179 articles were excluded after the evaluation of their full text.

## Results

### The most common chronic diseases associated with non-communicative older adults experiencing pain

Dementia was the most described disease associated with communicative inability in older adults experiencing pain or discomfort (2). Notably, orofacial pain was a highly prevalent type of pain, with an incidence ranging from 7.4 to 21.7%, especially in institutionalized older adult patients with dementia (48.8%) (24, 25). Impaired oral health care may be the result of executive cognitive dysfunction, motor apraxia, and/or abusing behavior such as neglect or resistance to care due to the patient’s behavioral disturbances (14, 25). Furthermore, suboptimal management of pain was found to be the catalyst for disruptive behavioral disturbances (26). In particular, verbalizations/vocalizations, sneezing, gasping, constrained facial expressions, restless or strained body expressions (e.g., raising the upper lip and guarding), impaired eating, agitation/aggressiveness, and resistance to care (2, 19, 27–30) were the most common disturbances displayed. As a result, the impaired ability to communicate made these vulnerable patients more likely to receive psychotropic medications than adequate pain medications (2).

Notably, verbalizations showed greater heterogeneity across different ethnic groups, potentially pointing out the socio-cultural background as a mediator of the overall pain experience (31).

While several articles demonstrate an improvement in pain recognition and its pharmacological management using observational scales in these patients, the clinical trial by Rostad and colleagues found that observational-based pain assessment in institutionalized patients with severe dementia did not result in an increase in analgesic drug administration (32–34).

Parkinson’s disease (PD) was the second most commonly reported disease associated with communicative inability and the experience of pain (35, 36). Although dementia was a comorbid condition in the advanced stage of PD, accounting for 30% of cases (37), poor communication was also associated with dysprosody, cognitive–linguistic impairment, alterations in social interactions, and pragmatics (37). Thus, an impaired ability to express pain through verbal and nonverbal communication (facial expression) was found in PD patients and casually linked to an impaired cognitive processing of painful sensations (35, 36).

Stroke was the third most described disease associated with poor communication of pain, with pain reported in these patients in a range of 42 to 72% (38, 39). In particular, 17.9% of patients had a co-occorrent diagnosis of dementia that was responsible for severe communicative inability, whereas aphasia or dysarthria were the main causes of non-communicative ability in those patients (40). In terminally ill patients with stroke, stroke-related pain was associated with central pain, shoulder–hand syndrome, and type 2 complex regional pain syndrome. Notably, pain behaviors were reported in 60% of dying patients, and wrinkled, contracted faces, moaning, and rubbing of the body were considered the most reliable pain indicators (39).

**Figure 2:**
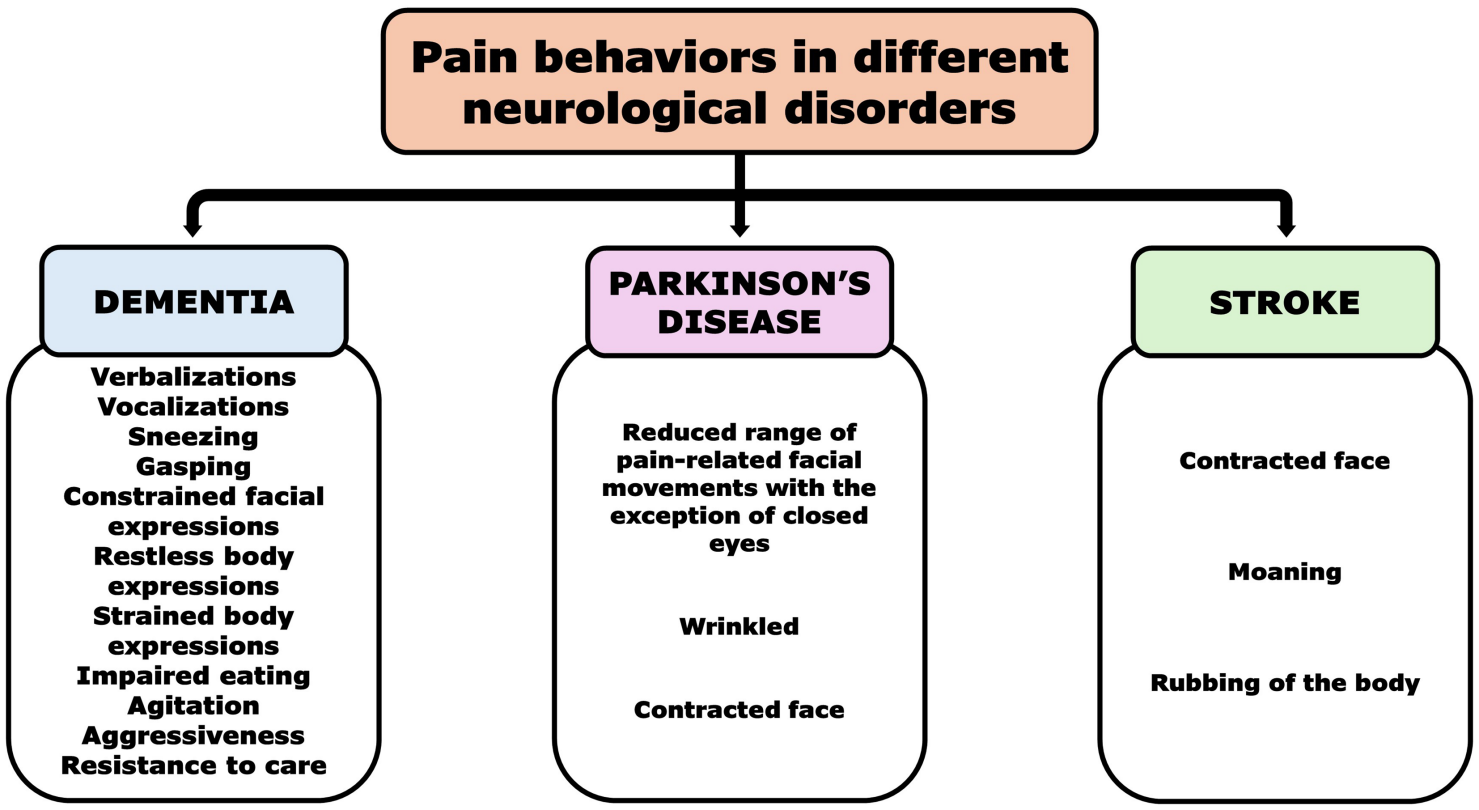
Pain behaviors in non-communicative patients with different neurological disorders [Dementia (2, 19, 27–30), Parkinson’s disease (36), and Stroke (39)].

Figure 2 summarizes the prevalent pain equivalents associated with neurological disorders in older adults with communicative inability.

### Diagnostic pain assessment in non-communicative older adults

The following are the main assessment tools present in the literature for assessing pain in the non-communicating patient, which have been developed especially for patients with dementia.

We propose to classify pain assessment scales according to the degree of expertise of users (as indicated by the corresponding tool validation studies), including healthcare providers (for example, staff members, nurses, nurses assistants), medical professionals, and inexperienced observers or raters. There are no instruments in our data that are restricted to family members’ use.

#### Pain assessment tools administered by healthcare providers, including trained personnel

DOLOPLUS 2: the Doloplus 2 is a tool based on the Douleur Enfant Gustave Roussy scale for young children, adapted for use in older adults (11). It evaluates three distinct pain equivalents: facial expression, psychomotor behaviors, and psychosocial behaviors. Administered by nurses, it takes 6–12 min to complete and has a score range of 0–30. A score of 5 out of 30 suggests pain (11, 41). It has adequate clinometric properties for patients with dementia but lacks information on clinical phenotypes and stages of dementia (11). Validated in both acute and long-term care settings, it is a valuable tool for assessing pain in older adults.

ALGOPLUS: Algoplus is a five-item scale consisting of different facial, body, and movement-related behavioral indicators (42). It takes a few minutes to complete, and a cut-off score of 2 out of 5 indicates pain. It has been found to have good inter-rater reliability, with high sensitivity and specificity for identifying pain in patients with dementia, depression, or both, potentially including a wider set of non-communicative disorders. This scale, in its validation in multiple languages (English, Spanish, Italian, Portuguese, and Turkish), has also been successfully applied to stroke patients (43). The tool has been validated in emergency departments, acute care (geriatric and non-geriatric), rehabilitation, and long-term care settings. The tool may be administered by healthcare providers, including medical personnel.

PAINAD: the Pain Assessment in Advanced Dementia (PAINAD) scale is a modification of available pain assessment tools (FLACC and DS-DAT) (44) developed through expert consultations (45). It is composed of five behavioral indicators, resulting in a total score ranging from 0 to 10 (11, 45). The scale requires a 5-min observation period and can be administered by a nurse or nursing assistant (46), after a training session (45). The PAINAD scale has been validated in persons with advanced dementia in both long-term care and acute geriatric wards. However, the small sample used in developing the PAINAD limits its findings (45). A study by Mosele and colleagues confirmed the reliability and feasibility of the scale compared to self-reported pain assessment methods (47). Cross-culturally adapted versions of the tool, such as the Korean version (PAINAD-K) (48) and the Turkish version (PAINAD-T) (49), have shown promising results in different healthcare settings, such as long-term hospitals (PAINAD-K) and palliative or intensive care units (PAINAD-T) (48, 49). Additionally, Pinto and colleagues provided a Brazilian Portuguese version of the scale (PAINAD-Brazil), which showed good validity, reliability, and reproducibility (50). Indeed, PAINAD-Brazil was validated in a variety of clinical settings with a broad spectrum of patients (aged 20 to 104 years), yielding similar results when compared to the original and European Portuguese versions of the PAINAD scale (50).

PACSLAC and PACSLAC II: the Pain Assessment Checklist for Seniors with Severe Dementia (PACSLAC) is a 60-item checklist that measures pain behavior in older adults with dementia (45). It covers four subscales related to facial expressions, body movements, social interaction and mood, and physiological circadian rhythms as pain equivalents (11). It takes 5–8 min to complete by a healthcare provider (e.g., nurse), and no score threshold is currently available (45, 51). Although the PACSLAC has shown good clinometrics in patients with dementia, it may not be effective in acute care settings for patients with limited communicative abilities (52). The PACSLAC, similar to PAINAD, was regarded as one of the most clinically valuable and psychometrically strong tools (53). A revised version, PACSLAC-II (54, 55) consists of 31 items and six pain assessment domains, as recommended by the American Geriatrics Society, aiming to overcome the limitations of the original version. It takes a few minutes to complete by trained nurses and has been validated in patients with moderate-to-severe dementia in long-term care facilities. It has also shown meaningful correlations with the original PACSLAC scores and the ability to differentiate between different pain-related clinical conditions. The scoring procedure is the same as in the original version (sum of the scores for the single items, scoring 1 if observed).

NOPPAIN: the Non-Communicative Patient’s Pain Assessment Instrument (NOPPAIN) is a nursing assistant-administered instrument consisting of four main sections (activity chart checklist, pain behavior presence, pain behavior intensity, and pain intensity). Properly, it collects information about pain behaviors and the conditions under which they are observed. Pain intensity is scored using a 6-point Likert scale (4, 11, 56, 57). The NOPPAIN takes an average of 8 min to be completed, requiring minimal training (4, 56). The total NOPPAIN score is the sum of the scores for the four subitems (range = 0–55) (57). Pain intensity is scored on an 11-point numerical rating scale, with different levels indicating severity of pain observed in the patient (56). A cut-off is not clearly established (45). The tool has been validated in persons with dementia in various settings (57), but its validity and generalisability may be limited due to the use of a video approach to simulate a painful situation. In a training setting involving 142 hospital staff members, Novello and colleagues validated an Italian version of the tool using five videotapes showing varying degrees of pain intensity. The findings revealed that the Italian version of NOPPAIN achieved significant construct reliability and inter-rater reliability, accurately identifying the varied levels of pain intensity (58).

ABBEY PAIN SCALE: the Abbey Pain Scale by Abbey and colleagues (11, 59) includes six behavioral indicators (59): vocalization, facial expressions, change in body language, behavioral changes, and physiological and physical change. It requires 1 min of administration by a trained nurse and provides a score for each pain equivalent (a total possible score ranging from 0 to 18). A score of ≥3 indicates pain, with specific ranges for mild, moderate, and severe pain (45). The scale could discriminate between types of pain (28, 59) and has been validated in patients with moderate-to-severe dementia residing in home care settings (45). Gregersen and colleagues (28) performed a cross-cultural validation of the Danish version of the Abbey Pain Scale in a hospital setting and in a large population of older adults with dementia. Notably, the tool was more sensitive for the assessment of acute pain than chronic pain, with potential applications in the breakthrough pain assessment of patients with dementia (28).

CNPI: the Checklist of Nonverbal Pain Indicators (CNPI) includes a six-item scale designed to measure pain behaviors in the geriatric population with cognitive impairment. It includes non-verbal vocalizations, facial grimacing or wincing, bracing, rubbing, restlessness, and vocal complaints (60). Completing the CNPI requires 5–10 min (11) and the semiquantitative score is based on the sum of the scores for the subitems both in resting conditions and after movement. A clear cut-off has not been established (60). Attention to pain-related behaviors during transfers and patient care activities requires a nurse’s administration of the tool (60). However, the CNPI has a dichotomous scoring method, which may limit its clinical utility as it does not accurately score the severity of pain (11, 61).

REPOS: the Rotterdam Elderly Pain Observation Scale (REPOS) consists of 10 behavioral items relating to face expressions, emotional states, bodily behavior, and vocalizations, which the observer scores as present (1) or absent (0) after a 2-min observation period, preferably during a possible painful moment of care. Total scores range from 0 to 10. A cut-off of 3 or higher, along with a proxy NRS score of 4 or higher, suggests an elevated likelihood of pain (62). Although the REPOS does not assess pain intensity, it incorporates a decision-making tree to assist observers in assessing scores and managing pain (62). During the validation phase in nursing home residents, a large correlation emerged between 10-item REPOS and PAINAD, while correlations with NRS (resident self-report and nurse’s NRS) were limited (62). Originally developed for nursing home residents, the REPOS was shown to be a reliable and valid instrument for chronic and subacute pain assessment in different settings (nursing home, hospital, palliative care center) and populations (nursing home residents with various cognitive levels, institutionalized adults with cognitive impairment, palliative care patients, and non-communicative hospital patients) (62, 63).

PADE: the Pain Assessment for the Dementing Elderly (PADE) consists of 24 items and covers three dimensions: facial expressions, activities of daily living, and the overall healthcare provider’s judgment of pain symptoms. It takes 5–10 min to be completed by trained personnel. Scoring procedures are somewhat inconsistent and heterogeneous, and no cut-off is clearly established. It has adequate clinometric properties for assessing patients with advanced dementia (11). Despite some limitations in a study conducted with residents of long-term care facilities with advanced dementia (64), the PADE was found to be a reliable and valid tool for assessing pain in older adults with advanced dementia in such settings (45).

ADD: the Assessment for Discomfort in Dementia (ADD) evaluates six behavioral pain equivalents (facial expression, mood, body language, voice, behavior, and others) in older adults in long-term care facilities and includes treatment interventions for pain and emotional distress. The ADD protocol led to a significant increase in the use of pharmacologic and nonpharmacologic comfort interventions (65–67), due to its interactive assessment, which allows nurses to assess and manage the unmet needs of people with advanced stages of dementia (67). However, there is no available scoring or rating for pain intensity.

FLACC: the Faces, Legs, Activity, Cry, and Consolability (FLACC) Observational Tool is a behavioral scale (68) used in the assessment of older adults with cognitive impairment residing in long-term care settings (65, 68). It consists of five items evaluated on a 3-point scale for a total range score of 0–10. Observation is provided by trained nurses. However, this scale has not been shown to be a reliable pain assessment tool in patients with cognitive impairment due to limited data and a lack of cut-off scores or feasibility evaluation (65, 69).

MOBID-2: the MOBID-2 Pain Scale (70) is the nurse-administered version of the MOBID Pain Scale with added items to assess musculoskeletal and visceral pain. It has been validated (71) in non-communicative patients with severe dementia across different settings (dementia-assisted living groups, long-term care units, rehabilitation units, and palliative care units). The tool requires about 4 min to be completed, and it is also suitable for the virtual assessment of pain. In 2022, Scuteri and colleagues translated, adapted, and validated the Italian version of the MOBID-2 Pain Scale (I-MOBID2), using psychometric testing of the MOBID-2 for non-verbal and severely demented patients (72). Two groups of trained nurses conducted a validation study on a small sample (*n* = 11), with an average execution time of 5.38 min. The results confirmed the psychometric properties of the scale, demonstrating that the I-MOBID2 is a valuable tool that may be further refined and employed in community settings with healthcare provider administration. Interestingly, the I-MOBID2 was selected as the pain assessment tool in a randomized, double-blind, placebo-controlled trial evaluating the efficacy of NanoBEO, an engineered bergamot essential oil with proven analgesic and anxiolytic properties, in reducing agitation and pain in advanced dementia patients (72).

#### Pain assessment tools requiring professional expertise (e.g., medical or expert personnel)

DS-DAT: the Discomfort Scale in Dementia of the Alzheimer’s Type (DS-DAT/DS-DAT modified) (44) assesses discomfort in older adults with advanced dementia of the Alzheimer’s type. This scale consists of nine items, measured after a 5-min observation period based on frequency, intensity, and duration. The total score ranges from 0 to 27, with higher scores indicating a high level of discomfort (44). The tool, originally developed for research, has been tested in several settings (65) and can be completed in about 15 min by an expert rater (65).

OPS-NVI: the Orofacial Pain Scale for Non-Verbal Individuals (OPS-NVI) (14) was developed to diagnose orofacial pain in non-communicative persons. It is a meta-tool of the PAIC (13), which evaluates facial activities, body movements, vocalizations, and specific oral behaviors. The score ranges from 0 to 10, and a score of ≥1 is suggestive of pain. It takes prior training provided by a hygiene care specialist to utilize the tool (14). The tool has been validated in persons with dementia and other non-communicative disorders in different settings [outpatient memory clinics, geriatric outpatient clinics, hospital nursing homes (14), as well as acute hospitals (25)].

MOBID: the Orofacial MOBID Pain Scale assesses the presence of orofacial pain or discomfort-related behaviors based on pain noises, facial expression, and reaction to care, as well as the presence of dementia-related behaviors such as anxiety, aggression, and confusion. It requires about 1 h of training and less than 5 min to be completed. The pain intensity is evaluated at rest and during each rated movement using the NRS, and the overall pain intensity score is rated using the same NRS quantitative scoring for each item (73, 74). The MOBID Pain Scale has been validated in older individuals with dementia with the use of video uptakes (74) and with increased reliability in repeated assessments. In line with this, Husebo and colleagues reported moderate-to-excellent intra- and inter-rater reliabilities of the pain intensity for each item as well as the overall pain intensity score in the MOBID Pain Scale (75). Originally, the MOBID Pain Scale was validated in a nursing home setting, wherein pain was assessed by an expert senior dentist who rated the video uptake of patients undergoing oral health care. The teeth/mouth care item was not included in the initial draft of the MOBID Pain Scale due to its limited correlation with the overall score. Toxopeus and colleagues aimed to assess the reliability of this item by reviewing teeth and mouth care video fragments with elderly care dentists. Notably, their findings showed that all consistent scores pertained to dementia-related behaviors, not to orofacial pain or disability-related behaviors, supporting the decision of Husebo and colleagues to exclude the teeth/mouth care item from the original MOBID version (76).

#### Pain assessment tools without requested or indicated professional training

PAINE: the PAINE assessment tool is a caregiver or informant rating scale (53, 65, 77) that consists of 22 items divided into two parts: in the first, the tool evaluates the presence of physical repetitive movements, vocal repetitive behaviors, unusual behaviors, and any involvement in activities; in the second part, it explores physical signs of pain (in a dichotomous mode, yes/no) (77). The scale must be administered by an observer familiar with the patient, and in a study by Cohen and colleagues (77), data was collected from direct-care staff members. While it has been shown to be correlated with the PADE score, self-report, and observation (67), there is no information available on cut-off scores, rater training, or the feasibility and clinical utility of the tool (65).

PAIC: the Pain Assessment in Impaired Cognition (PAIC) scale was developed in a multidisciplinary effort to offer a scale that can be used by medical professionals (nurses, doctors). It is a meta-tool based on existing instruments with 36 consecutive items clustered into the domains of facial expression, vocalization, and body movements (13). This is an internationally agreed-upon tool to assess pain in individuals with cognitive impairment, especially dementia. All relevant pain-related observational items had been identified. However, existing scales include pain-irrelevant items or items of poor psychometric quality. Therefore, the main task was to reduce and refine the number of items (78). The PAIC scale has been validated in patients with dementia living in nursing homes. Each item is scored on a 4-point scale, and an observation time of at least 3 min in different settings is recommended (78). Observations can be conducted by healthcare professionals without receiving any special training (78). However, a standardized cut-off is unavailable. Four additional items (pained expression, raising the upper lip, pain-related words, and guarding) have been validated, and the item gasping has been identified as specific to pain (13).

The Observational Assessment of Pain or Distress tool has been used in post-acute care facilities with non-communicative older adults with dementia, showing an association between setting type and pain or distress (19). It is completed by staff observation in three steps: evaluating the patients’ ability to participate in a pain interview, the presence of potential pain indicators, and the response to pain treatment.

No standardized observation-based tool for the clinical assessment of pain in non-communicative patients with PD is available. Also, there is no observational tool specifically studied for the condition of stroke.

The Australian Pain Society’s Management Strategies of Pain in Residential Aged Care Facilities emphasizes the importance of using the observational pain measures we described both at rest and during movement (e.g., during transfers) to detect any exacerbation of the possible underlying pain condition (7, 8, 79, 80).

Lastly, we point out that NRS (or verbal descriptors) can be used by individuals with mild to moderate cognitive impairment and borderline communicative capacity, while observational scales are preferable in more advanced stages of cognitive impairment. The National Guidelines on the Assessment of Pain in Older People in the United Kingdom and the Australian Pain Society both support these recommendations (7, 79, 80). For situations relevant to our study, like limited indications of stroke and communication problems related to Parkinson’s disease, there is no specific guideline (7, 8, 79, 80). Table 1 summarizes the above-mentioned major findings.

**Table 1:** Description of the main observational pain tools for non-communicating older adult patients (3).

Observation-based tool	Setting	Scoring	Time and mode of administration	Description of the tool	Type of population	Outcomes of interest
**Pain assessment tools administered by healthcare providers, including trained personnel**						
DOLOPLUS 2 (11, 41, 45, 65, 81)	Acute and long-term care settings	Binary scores are summed up Score ranges: 0–30 Cut-off: 5/30 (suggestive of pain)	6–12 min by a trained nurse	3 distinct pain equivalents (facial expressions, psychomotor, and psychosocial behaviors)	Cognitively impaired older adults (a few are non communicative) Clinical phenotype and stages of dementia in patients are partially reported	Inaccurate for clinical use because of its low reliability
ALGOPLUS (42, 43)	Emergency departments Acute care (geriatric and non-geriatric) Rehabilitation Long-term care	Cut-off: 2/5 (indicative of pain)	About 1 min to be completed Trained nursing and/or medical staff for administration	5-item scale (facial, body, and movement-related behavioral indicators)	Patients with dementia or depression	Accurate for clinical use (mean Algoplus score reduction) after starting pain management
PAINAD (7, 11, 45–50, 82–84)	Long term care Acute geriatric care LTC hospitals Palliative or ICU	The total score ranges: 0 (no pain) – 10 (maximum pain) Cut-offs are not reported No score threshold: qualitative scoring system (the highest score indicates more severe pain)	5 min for observation Nurse or nurse-assistant administration A training session is required	5 items (breathing, vocalization, facial expression, body language, and consolability) rated on a 3-point scale	Patients with advanced dementia.	The original work of Zwakhalen was based on a small sample, limiting the findings A further attempt confirmed the psychometric properties of PAINAD, by comparison with NRS PAINAD is a sensitive tool for detecting pain in adults with dementia but does have a high false positive rate The Brazilian version of the tool proved to be useful in daily routine care of hospitalized adult and elderly patients in a variety of clinical settings
The Pain Assessment Checklist for Seniors with Severe Dementia (PACSLAC) (11, 45, 51, 52)	Long-term care	Score ranges: 0–60 No score threshold is currently available	5–8 min to be completed by healthcare providers (nurses).	60-item behavioral scale	Older adults with dementia and/or limited communicative abilities Over 65-year-old patients with hip fracture	Useful for monthly or quarterly clinical pain assessments
PACLASC II (7, 54, 55)	Long-term care	Total score is the sum of the scores for single items (scoring 1 if item is observed) No cut-off is available	Few minutes to be administered by trained nurses	31 items, 6 behavioral indicators (facial expressions, verbalizations and vocalizations, body movements, changes in interpersonal interactions, changes in activity patterns or routine, and changes in mental status)	Patients with moderate to severe dementia	Accurate for clinical use of pain assessment Differentiation between pain-related clinical conditions, minimizing overlap with behaviors that also occur in nonpainful situations It does need a short form and more testing in larger scale studies
NOPPAIN (4, 11, 45, 56–58)	Nursing home General Medicine Geriatric acute Wards	The total NOPPAIN score is the sum of the scores for the 4 subitems (range = 0–55) Pain intensity scoring: 0: absence of pain; 1–3: mild; 4–6: moderate; 7–9: severe; 10: more severe pain The cut-off is not clearly established	8 min to perform by untrained healthcare provider	4 sections 6 items (pain words, pain noises, pain faces, rubbing, bracing, and restlessness) 2 dimensions of pain evaluated: presence (yes/no), intensity (NRS 0 – none – to 5 – worst).	Patients with mild to moderate dementia	Accurate for clinical use and daily pain assessment It is considered a preferable tool in a nursing home setting Limited validity and generalizability because developers acted out a painful situation (using a video approach) The combination of text and pictures makes the tool easier to understand
Abbey Pain Scale (11, 28, 45, 59)	Home care setting Residential aged care facilities Hospital Nursing homes	Each item is evaluated on a 0–3 scale (0 = absence, 3 = severe expression) Scoring ≥3 indicates pain Score ranges for pain intensity: 0–2 absence; 3–7 mild, 8–13: moderate 14–18: severe	1 min to be completed Trained nurses administration	6 items: vocalization facial expressions change in body language behavioral changes physiological change physical change	Subjects with moderate to severe dementia	It could discriminate the type of pain (such as chronic, acute, or acute superimposed on chronic pain) Accurate for daily clinical pain assessment (breakthrough pain)
Checklist of Non-Verbal Pain Indicators (CNPI) (11, 45, 60, 61)	Acute hospital setting	Semiquantitative scoring method Scoring range: 0–6 No clear cut-off scores to indicate severity of pain	It takes 5–10 min to be completed Nurse-assistants administration At rest and under movement evaluation	6-item scale: non-verbal vocalizations facial grimacing or wincing bracing rubbing restlessness vocal complaints	Cognitively impaired older adults	Acceptable clinometric properties Dichotomous scoring method reduces sensitivity and fails to appropriately score the severity of pain, limiting its clinical use and daily pain assessment
The Rotterdam Elderly Pain Observation Scale (REPOS) (62, 63)	Nursing home. Hospital. Palliative care center.	Total score ranges: 0–10 Cut-off ≥3 suggests pain (chronic or subacute) pain.	2-min observation period (at rest and in a potentially painful situation). In nursing homes: caregiving nurses administration.	10 behavioral items. Dichotomous scoring system (0 absent, 1 present).	Patients with different levels of cognitive impartment. Non-communicative hospital patients. Palliative care patients. Institutionalized adults with cognitive impairment,	In the original version, high correlation emerged between REPOS and PAINAD (low correlations were found between REPOS and NRS-resident and NRS-nurse). A step-by-step decision tree is provided to aid in score interpretation and pain management. Its conciseness suggests feasibility in daily practice
Pain Assessment for the Dementing Elderly (PADE) (11, 16, 45, 64, 65)	Long-term care facilities	Inconsistent and heterogeneous scoring procedures No cut-off is clearly established	5–10 min by trained personnel.	5 items (AGS guidelines)	Older adults, with advanced dementia (*n* = 25 study 1; *n* = 40 study 2)	Inaccurate for clinical use because of its low reliability Different rating systems are used in the same tool (Likert scales/VAS) Likert scales/binary scores
Assessment for Discomfort in Dementia (ADD) (7, 65)	Long term care facilities	No available scoring or rating for pain intensity	Nurse-administered intervention	Discomfort and pain assessment tool Six behavioral pain equivalents (facial expression, mood, body language, voice, behavior, and others)	People with moderate to severe dementia	Complexity in administration makes the tool time-consuming and not suitable for daily clinical practice
FLACC (17, 65, 68)	Long term care facilities	Total range score: 0–10 No cut-off scores are reported	Trained nurses observation.	Behavioral scale 5 items (face, legs, activity, crying, and consolability) on a 0–3 points scale each	Small sample (*n* = 6)	Designed for use with children Clinical usefulness of the tool in older adults remains unknown Unhelpful pain assessment tool for cognitively impaired older adults
Mobilization-Observation-Behavior-Intensity-Dementia (MOBID-2) (70–72)	Dementia-assisted living groups Long-term care units Rehabilitation unit Palliative care unit	No established cut off score	Average of 4.37 min for administration Nurse-administered	Extended two-part version of the MOBID Pain Scale Key indicators of pain behavior include pain noises, facial expression, and defense 10 items, 5 per part	Non-communicative patients with severe dementia	Accurate for clinical use for daily pain assessment Suitable for the virtual assessment of pain
						I-MOBID2 was selected as the pain assessment tool to evaluate the efficacy of NanoBEO, a specially formulated bergamot essential oil, in reducing agitation and pain in advanced dementia patients The validated Italian version of MOBID-2 Pain scale (I-MOBID2) had an average administration time of 5.38 min
**Pain assessment tools requiring professional expertise (e.g., medical or expert personnel)**						
DS-DAT (Discomfort Scale in Dementia of the Alzheimer’s Type) (44, 65)	Nursing homes Long-Term care facilities Hospitals Veteran Administration facilities	Score 0–3 points, the total score from ranges 0–27	5-min observation period before administration, according to 3 variables (frequency, intensity, and duration) It requires well-trained raters	Modified tool, from the original of Hurley and colleagues Properly, discomfort assessment tool. 9 items (2 positive, 7 negative)	Patients with Alzheimer Disease	Time-consuming scoring system (especially the scoring of intensity and duration of discomort) limits the feasibility of the tool Treatment protocols for discomfort (measured with this tool) are different from those for pain, which is not measured with this tool
Orofacial Pain Scale For Non-Verbal Individuals (OPS-NVI) (13, 14, 24, 25, 85, 86)	Outpatient memory clinics Geriatric outpatient clinic Hospital. Nursing homes Acute hospitals	Total score ranges: 0–10 Cut-off ≥1 indicates pain	At rest and during activities (drinking, chewing, oral hygiene care) observation Trained observers administration: training in the use of the OPS-NVI by one of the developers (expert dentistry)	Meta-tool of PAIC (Pain Assessment in Impaired Cognition) 4-item observation (facial activities, body movements, vocalizations, specific oral behaviors)	Persons with dementia and other non-communicative disorders	Accurate for orofacial pain assessment in non-communicating patients
Orofacial Mobilization–Observation–Behavior–Intensity–Dementia (MOBID) Pain Scale (71, 74–76)	Nursing home	Unclear scoring instructions Evaluation of pain intensity: at rest and in movement using NRS No cut-off is mentioned	1 h for training <5 min for administration Expert dentist assessment (during videotapes of patients undergoing oral care)	Assessment of behavior (pain/discomfort or dementia related), 0–3 scale	Patients with severe cognitive impairment	Insufficient clinical evidence for accurate pain assessment Teeth/mouth care item was excluded from the tool’s initial draft due to its limited correlation to the overall score
**Pain assessment tools without a requested or indicated professional training**						
PAINE (53, 65, 77)	Nursing homes	No information about cut-off scores	Assessment is done on a 0–7-point frequency scale over the previous 2 weeks	Caregiver/informant rating scale 22 items on pain-related behaviors (facial expressions, verbalizations, body movements, and changes in activity patterns or routines)	Noncommunicative older adults, with dementia	Missing data (raters’ training, feasibility, and clinical utility of the tool) and the few available limit the considerations about clinical utility
PAIC15 (13, 78)	Nursing homes	0–4 point scale for each item (0 = not at all, 1 = slight degree, 2 = moderate degree, and 3 = great degree) A standardized cut-off is missing	Observations provided by healthcare professionals without any special training 3-min observation period (at rest and during daily living activities) is recommended	Meta-tool of PAIC (Pain Assessment in Impaired Cognition) 15 behavioral descriptors 5 behavioral indicators for each behavioral category (facial expressions, body movements, and vocalizations)	Persons with dementia	The tool validated four other items (‘pained expression’, ‘raising upper lip’, ‘pain-related words’, ‘guarding’) and the item ‘gasping’ as pain-specific
Observational Assessment Of Pain Or Distress tool (19)	Post-acute care (PAC)	No cut-off is mentioned	Staff observation	3-step observation of pain/distress indicators over 3 consecutive days (1) Behavioral indicators (2) Frequency (3) Resolution or reduction after pain medications	Non communicative patients	Difficulty in assessing the population with cognitive impairment and mood, as challenging factors in evaluating pain and other needs in these selected patients

### High-technology tools for pain assessments

Recently, El Tallawy and colleagues (87) showed promising solutions offered by high-tech tools for pain assessment, especially in patients affected by moderate to severe dementia. The new technologies rely on various tools, including the detection of facial expressions, facial muscle movements, vocal cord responses, and behavioral changes caused by pain. The Automatic Pain Assessment with Video Systems is suitable for older patients with dementia and can complement other pain assessment methods. This tool primarily emphasizes the automatic analysis of facial expressions. Another promising high-tech tool is the smart wearable shirt, which is able to continuously monitor human physiological signs (heart rate, any changes in respiratory function, body movements) without impacting daily living. Finally, the authors refer to smart homes: residences equipped with Internet-connected devices used to collect, transfer, store, and analyze data over a network. Such tools would be able to minimize the possible bias found in other clinical methods and improve the quality of pain assessment.

## Discussion

Our findings underscored that dementia, PD, and stroke were the most frequently described neurological diseases associated with communicative inability in older adults experiencing pain. To date, most evidence underscores that moderate-to-severe dementia stages have a major impact on the ability to express pain, increasing the risk of inappropriate medication prescriptions and poorer quality of care (2, 3, 48, 70, 88–91).

All these neurological conditions could affect not only pain communication but also its processing. In particular, it is noteworthy that pain processing undergoes substantial alterations in the presence of neurodegenerative diseases associated with dementia, and namely, incoherent pain-related facial expressions in response to pressure stimuli may be associated with structural changes in the prefrontal areas, the loss of inhibition of pain stimuli, and the amplification of the overall pain response in those patients (92). Similarly, these vulnerable patients experience altered descending endogenous pain modulation that impacts their ability to appropriately report pain (93–96).

Notably, alterations in the processing of painful stimuli have also been reported in different types of dementia, and, in particular, patients with vascular dementia were deemed to experience hyperpathia, whereas those with frontotemporal dementia seemed to experience reduced pain cognition (35, 97).

Our findings also originally emphasized that orofacial pain is one of the most painful conditions (24, 25), ranging from 7.4 to 60% in older people with dementia (98, 99), suggesting that the adoption of adequate oral health care may turn out to be a key relevant measure to prevent behavioral equivalents of pain in such vulnerable patients.

Moreover, on the basis of our results, a cluster of behavioral equivalents in patients with dementia experiencing pain was described, including verbalizations, gasping, constrained facial expressions, guarding, and restless or strained body expressions (13, 100).

Similarly, Ford and colleagues (31) have identified rubbing, bracing, restlessness, and pain vocalization as the most reliable behavioral equivalents of pain in patients with dementia.

However, the identification of pain in patients with communicative inability should go beyond the metrics of dementia, including different neurological conditions that may share similarities in terms of atypical presentation, underdiagnosis, and undertreatment.

Notably, regarding PD, pain is considered a relevant non-motor symptom in this condition, increasing the disease burden and affecting the quality of life (101). In particular, hypernociception may precede the development of the motor symptoms, and chronic pain may be considered the most prevalent non-motor symptoms of PD (102). Priebe and colleagues (36) have underscored that patients with PD experience a reduced range of pain-related facial movements in response to a pain trigger. However, facial movements with the eyes closed were unaltered, suggesting that ‘eye closure’ may be considered a reliable pain equivalent in those patients. Additionally, the overall frequency and intensity of facial movements in response to pain stimuli were reduced in patients with PD experiencing the ‘off phase’. This is a clinical phase of motor and non-motor downregulation due to long-term levodopa administration (36), suggesting that the dimension of pain may be associated with the extent of dopaminergic deficiency and related fluctuations (103).

As already underlined, the ‘eye closure’ could be a behavioral equivalent in patients affected by PD (36), although no specific tool has been validated in this type of neurodegenerative disease to estimate pain if there are communication issues, leaving a gap of knowledge (104). Relatively recent findings have implemented the classification and diagnosis based on the PD-Pain Classification System (103), which enables the differentiation of PD-related pain into nociceptive, neuropathic, and nociplastic types (103). Although the system has definitely improved the mechanistic understanding of pain in PD, pain assessment in patients with cognitive impairment and/or communicative inability is still largely unaddressed.

Stroke was the third most described clinical condition associated with communicative inability and pain. The presence of aphasia can affect speaking or auditory comprehension, and similarly, dysarthria may affect speech articulation for muscle coordination, making speech intelligibility possibly impaired. Although the multifaceted origin of the altered communication ability in patients with stroke is reported, evidence is conflicting regarding whether the assessment of patients with stroke and aphasia could rely on self-reported pain instruments. Mandysova and colleagues (105) concluded that a major concern that permeates several studies is the fact that stroke with severe communication problems fails to be appropriately diagnosed with self-report tools, and the majority of studies have focused mainly on mild-to-moderate aphasia. The use of the PACSLAC-II along with self-instruments for such a vulnerable population is then recommended (54, 106). Moreover, in their retrospective study on terminally ill patients with stroke, Mazzocato and colleagues (39) underscored a cluster of pain equivalents, such as wrinkled, contracted faces, moaning, and rubbing, which may be a preliminary platform for future studies to bridge the gap in knowledge.

Relative to the tools currently in the literature for pain assessment in nonverbal patients, it is beyond the scope of this review to give guidance on the most indicated one.

In a recent systematic review, strong and moderate evidence supported the use of the Facial Action Coding System, PACSLAC, PACSLAC-II, CNPI, Doloplus 2, Algoplus, MOBID Pain Scale, and MOBID-2 Scale for the assessment of pain among patients with dementia. However, insufficient time to use measurement tools, protracted time of administration and interpretation of results, undertreatment of pain in people with dementia, fear of side effects or drug interactions, limited evidence of the responsiveness, structural validity, and measurement error of the identified measures confine the use of most of the observational tools in research and point out the need for multidimensional tools (107).

Recently, the PAIC15 tool was developed to frame a multi-component pain assessment of patients to optimize the discrimination between normal and abnormal and/or noxious behaviors in patients with cognitive impairment and to differentiate acute from chronic pain in older adult patients with dementia (107). Hadjistavropoulos and colleagues (53) emphasize the need for a multi-component approach for pain assessment in non-communicative older adults, underlying the need to assess pain under movement and considering assessments before and after interventions.

However, the validity of the above-mentioned scales in the context of other neurological disorders remains a matter of debate, and further research is then needed to validate tools for clinical conditions other than dementia and in different clinical settings. In fact, as could be inferred, there are substantial differences in the types of pain that can be developed and the ways in which it is processed in subjects with these clinical conditions. By virtue of this, pain behaviors may be partially different in each condition, not completely allowing the rating scales for dementia to be generalized to other diseases as well. This is because most of the instruments were developed in patients with cognitive impairment, with some scales studied in patients with stroke but not specifically in patients with PD. This review can provide a starting point for the shared pain behaviors of the different diseases to begin with a tool that can be applied apart from dementia.

Another important point to highlight about the tools available for this issue is that they are mostly designed with dichotomous logic. This approach does not fully enable clinicians to understand the severity of the pain or hypothesize its nature. Moreover, this dichotomous logic complicates therapeutic management, as there are no standard criteria for initiating or revising therapy.

Furthermore, according to the main guidelines on this topic, the NRS, or verbal descriptors, can be used to assess pain in patients with mild-to-moderate neurocognitive disorders, as their ability to express pain through these methods is generally preserved (7, 8, 79, 80). Therefore, these tools can be a first choice in such conditions, while observational tools are mainly dedicated to patients with severe cognitive impairment. However, the complexity of daily clinical practice with these patients must be considered, given their possible fluctuations in cognitive status, especially in certain subtypes of neurodegenerative disorders, such as dementia with Lewy bodies, and possible incident cases of delirium (108, 109). Additionally, there is little evidence providing specific guidance in this sense for patients with communication problems associated with PD and stroke. Therefore, it is essential to conduct more studies on these topics with large sample sizes, considering all these issues, to make pain assessment and management for these patients increasingly systematic and effective.

To date, increasing education and research are still needed to minimize barriers and optimize a gold standard assessment tool for pain in non-communicative patients that should ideally include a multidimensional construct to address the complexity of this vulnerable and frail population (53, 107). Another opportunity to make the identification and management of pain in the non-communicating patient more cross-cutting and feasible is offered by new technologies. In particular, tools that take advantage of artificial intelligence may certainly be useful in the near future to make the assessment of indirect signs of pain more systematic and objective (87). However, these technologies still need much validation and optimization to enter everyday clinical practice. Furthermore, in order to overcome the current limitations of pain assessment in the non-communicating patient, it is crucial to ensure that this new field is not restricted solely to assessing pain in dementia but should also encompass the other neurological disorders discussed previously. New technologies, in the broader context of telemedicine, could make it possible to make the assessment of pain in neurological disorders described above more widespread, as is already happening, for example, with regard to telerehabilitation in entirely similar disease scenarios (110, 111). However, more research is needed even at this level.

The limitations of the present study include the lack of clinical phenotypes of patients, such as frailty or multimorbidity, which were not systematically investigated. Additionally, the heterogeneity of settings in terms of standardized requirements and facilities, as well as the limited population sample and the low number of prospective studies, were other sources of variability.

The strengths of the study are in its methodology, which is based on the SANRA and maintains a narrative approach to the presenting findings. In addition, the study presents evidence suggesting that non-communicative pain assessment may be applicable to other neurological diseases beyond dementia.

## Conclusion

The present narrative review provides an update on the prevalent diseases beyond dementia associated with a communicative disability and a painful condition in older adults.

Standardizing methods for assessing pain in clinical settings is crucial, with a focus on using patient self-report tools whenever possible and observational scales when self-reporting is not feasible, as evidenced by multiple clinical recommendations (17, 18, 53).

The rapidly aging population carries a growing number of neurological conditions that share communicative disabilities; thus, the mandatory issue of early identification of pain in such a vulnerable population to constrain unfavorable clinical outcomes and reduced quality of life is a top priority. Alongside improving professionals’ training, education, and empowerment (38, 112, 113), the implementation of technology, such as specialized software capable of assessing pain levels concurrently, offers a promising integrated solution that warrants further exploration in the future (106, 114–119).
